# Arterial stiffness and collagen expression in mice lacking NO-sensitive guanylyl cyclase

**DOI:** 10.1186/2050-6511-16-S1-A46

**Published:** 2015-09-02

**Authors:** Sarah Dünnes, Dieter Groneberg, Volker Herold, Peter Jakob, Andreas Friebe

**Affiliations:** 1 Physiologisches Institut, Universität Würzburg, Würzburg, Germany; 2 Lehrstuhl für experimentelle Physik 5, Universität Würzburg, Würzburg, Germany

## Background

NO-sensitive guanylyl cyclase (NO-GC) is the most important receptor for NO. The NO signal is transmitted through an increase in cGMP to act on several effector molecules. The NO/cGMP cascade is involved in the regulation of vessel diameter ultimately contributing to set the blood pressure. Lack of NO or NO-GC is thought to alter the responsiveness of blood vessels as well as their composition. We hypothesize that arterial stiffness may be altered in the absence of NO-GC.

## Methods

As arterial stiffness is an import indicator of cardiovascular risks and mortality, we aimed at determining the role of NO-GC for aortic stiffness using general KO mice (GCKO) and smooth muscle-specific KO mice (SMC-GCKO) for NO-GC. Measurement of pulse wave velocity (PWV) was frequently used to determine aortic stiffness. We used magnetic resonance tomography (17.6 Tesla) for non-invasive measurement of local aortic PWV. PWV was calculated by simultaneously measuring of cross-sectional change and volume flow in a defined time.

## Results

Preliminary data indicate an increased PWV in GCKO mice whereas in SMC-GCKO, stiffness is unaltered. Aortic diameter was reduced in GCKO compared to WT whereas that in SMC-GCKO was not significantly different from controls. Global deletion of NO-GC did not affect cardiac output. These data will be correlated to structural data of aorta from these strains.

## Conclusion

Deletion of NO-GC affects the physical properties of vascular tissue. This effect appears to be independent of hypertension.

